# Understanding the menstrual hygiene management challenges facing displaced girls and women: findings from qualitative assessments in Myanmar and Lebanon

**DOI:** 10.1186/s13031-017-0121-1

**Published:** 2017-10-16

**Authors:** Margaret L. Schmitt, David Clatworthy, Ruwan Ratnayake, Nicole Klaesener-Metzner, Elizabeth Roesch, Erin Wheeler, Marni Sommer

**Affiliations:** 1 Mailman School of Public Health, Columbia University, New York, NY 10032 USA; 2 International Rescue Committee, 122 W. 42nd Street, New York, NY 10168 USA; 3 Mailman School of Public Health, Columbia University, New York, NY 10032 USA

**Keywords:** Menstruation, Menstrual hygiene management, Myanmar, Lebanon, Humanitarian response

## Abstract

**Background:**

There is a significant gap in empirical evidence on the menstrual hygiene management (MHM) challenges faced by adolescent girls and women in emergency contexts, and on appropriate humanitarian response approaches to meet their needs in diverse emergency contexts. To begin filling the gap in the evidence, we conducted a study in two diverse contexts (Myanmar and Lebanon), exploring the MHM barriers facing girls and women, and the various relevant sectoral responses being conducted (e.g. water, sanitation and hygiene (WASH), Protection, Health, Education and Camp Management).

**Methods:**

Two qualitative assessments were conducted: one in camps for internally displaced populations in Myanmar, and one with refugees living in informal settlements and host communities in Lebanon. Key informant interviews were conducted with emergency response staff in both sites, and focus group discussion and participatory mapping activities conducted with adolescent girls and women.

**Results:**

Key findings included that there was insufficient access to safe and private facilities for MHM coupled with displacement induced shifts in menstrual practices by girls and women. Among staff, there was a narrow interpretation of what an MHM response includes, with a focus on supplies; significant interest in understanding what an improved MHM response would include and acknowledgement of limited existing MHM guidance across various sectors; and insufficient consultation with beneficiaries, related to discomfort asking about menstruation, and limited coordination between sectors.

**Conclusions:**

There is a significant need for improved guidance across all relevant sectors for improving MHM response in emergency context, along with increased evidence on effective approaches for integrating MHM into existing responses.

## Background

Over 30 million girls and women are currently displaced due to conflict and disasters across the world; a record high since World War II [1]. A common and significant challenge they face is the ability to manage their menstruation safely, comfortably and with dignity. In many emergency contexts, women and girls lack access to basic materials, such as sanitary pads, cloths and underwear, that are needed to manage monthly blood flow [2–4]. Privacy is often non-existent while in transit, or in camps or informal settlements [4–6], and they often lack easy access to toilets, which even if available, may lack doors, locks and lighting and are inadequate to manage menses. Access to water and places to wash and dry reusable pads and cloths, or to dispose of used materials are often scarce [2, 7]. Such factors can increase their risk for exposure to violence and exploitation, particularly at nighttime when seeking out private spaces to manage sanitary needs [8]. These access challenges faced by displaced girls and women can occur in a range of contexts, including both higher and low income countries and in rural, camp and urban settings. These barriers may be intensified by cultural beliefs and taboos, in addition to the challenges associated with the  social dynamics among women in lower-income contexts that might lead them to be less likely to demand improved services or supplies for such a taboo issue.

A significant challenge to addressing MHM barriers in emergencies is the on-going secrecy, shame and taboo that frequently surround menstruation, hindering adequate assessment and identification of contextually appropriate solutions [9–11]. Girls and women hesitate to speak openly about menstruation, and emergency response staff may feel ill equipped to explore the topic. Cultural beliefs frequently influence menstrual practices, such as the types of materials that girls and women use, their methods for disposing of menstrual waste (e.g. burying versus communal trash bins) [3, 10, 12], and their preferences for how and where to wash and dry reusable materials [7, 13].

A prior review that was updated in 2016 found similar, stark findings regarding existing documentation, guidance materials, and the perspectives of humanitarian response practitioners on MHM in emergencies [14, 15]. The findings revealed a lack of uniform guidance for incorporating attention to MHM into various sector responses, including the types and timing of programmatic activities (e.g. inclusion during acute versus later phases of emergencies). Existing guidance that does mention MHM is often limited and concentrated within the water, sanitation and hygiene (WASH) sector. Yet in order to reach women and girls, an MHM response should be cross-sectoral in order to better address the demand side (e.g. Protection, Health, Education), with the Health sector, for example, needing supplies for bleeding management and appropriate WASH facilities at health clinics, and the Education and Protection sectors requiring female friendly toilets for girls and female staff, and emergency supplies of sanitary pads and underwear. The reviews also revealed the existence of numerous internal organizational dialogues over how to improve future MHM response, and a gap in documented evaluation, including lessons learned, of existing MHM programming, and insufficient assessment of beneficiary experiences.

The assessments were part of a larger project aimed at expanding the evidence and guidance on MHM during emergencies that was initiated in 2015 by the International Rescue Committee (IRC) and Columbia University’s Mailman School of Public Health [16]. Along with adding to the evidence, the project is developing an MHM in emergencies toolkit in partnership with the humanitarian response community that is being piloted and will be launched in 2017. To begin filling the gap in evidence and guidance on MHM in emergencies, qualitative assessments were conducted in two diverse humanitarian response settings in Myanmar and Lebanon. The primary objective was to identify key barriers to MHM among displaced adolescent girls and women, and to what degree each response addressed MHM needs. The secondary objective was to generate insights into the types and content of MHM guidance that would improve coordination and response in future emergencies.

## Methods

### Study setting

The study was conducted in two humanitarian populations receiving health and/or gender-based violence (GBV) and protection services from the IRC: internally displaced persons (IDPs) in camps in Rakhine State, Myanmar including in Buddhist and Muslim populated camps, and Syrian Muslim refugees living in host communities or informal settlements (e.g. tents and makeshift structures) in urban and peri-urban locations across Lebanon (Tripoli, Beirut and the Bekaa Valley). The IDP camps in Myanmar, located in rural, flood prone areas, included basic shelters for housing with local markets, health and education infrastructure. In Lebanon, the refugee population acquired their own housing (formal or informal), and primarily accesses these services alongside the local population in Beirut, Tripoli and Bekaa Valley. Some additional social services (health and protection) are being provided by local and international NGOs for the Syrian refugee population. In both locations, IRC provided protection programming for adolescent girls and women, in addition to a range of other services including health, NFI’s and WASH (Myanmar only). As in most emergencies, other humanitarian agencies covered other sectors such as shelter, education, WASH and nutrition.

### Research design and methods

Qualitative research methods were utilized in each context to assess adolescent girls’ and women’s experiences with menstruation, and that of emergency staff responding to meet their needs. The staff included both international and local (in-country) NGO personnel of both genders and across a range of management levels. Those working on protection issues with women and girls in particular were a range of ages. Research methods included key informant interviews (KIIs) conducted with staff from different sectors and organizations, focus group discussions (FGDs) with adolescent girls and women, and participatory mapping (PM) activities with adolescent girls. The focus of the KII guides included attention to MHM within the response, timing and content of MHM activities, implementation challenges, sector-specific considerations, best practices or lessons learned, and recommendations for the development of the MHM in emergencies toolkit under development. The FGD guides included questions regarding girls’ and women’s cultural beliefs surrounding menstruation, methods or barriers to accessing menstrual materials, where and how they manage their menstruation in the context (including challenges they face), gaps in knowledge or information available on topics related to MHM, and feedback and advice on how to better support girls and women MHM in their respective contexts. The PM activity was conducted with groups of 8–10 adolescent girls in small groups (2–3 per group) who were asked to develop a map depicting their immediate communities. Next, they were asked to identify locations on the map where they manage their menstruation (i.e. for changing, disposal, washing and procuring materials), and to indicate areas on the map where they felt safe/unsafe and comfortable/uncomfortable managing menses, with an explanation provided for these perceptions.

### Sample and recruitment

The sample (see Table 1) for the KIIs included a range of cross-sectoral humanitarian staff (male and female) from numerous humanitarian response organizations operating in each context. Key informants (*n* = 17) were sampled purposively; maximum variation sampling was used to ensure at least two individuals were sourced from each sector (WASH, Education, Health, Protection, Camp Management). The sample for the FGDs included adolescent girls and women between the ages of 14–49 years. Purposive sampling methods were utilized to ensure variation across ethnicity, age, and living situation (e.g. camp, informal settlements and host communities). The groups were stratified into three age groups (14–18; 19–25; 26–49 years of age). The age stratification was aimed at increasing the comfort and participation of girls and women. A total of 2 FGDs per age group per country (*n* = 6; total *n* = 117) occurred in each country. The mapping activity was conducted with two separate groups (*n* = 39) of adolescent girls (14–18 years of age) in each country.

**Table 1:** Number of participants

	Lebanon	Myanmar	Total
Key Informant Interviews with emergency response staff	8	9	17
Focus Groups Discussions with women (aged 19–25; 26–49)	53	64	117
Focus Groups Discussions with adolescent girls (aged 14–18)	14	18	32
Participatory Mapping with adolescent girls (aged 14–18)	16	23	39

Data collection occurred over a 2-month period in September and October 2015. The research team included female staff from Columbia University (CU) and the IRC. All activities were conducted in a confidential setting, with female facilitators and translators of Myanmar and Lebanese descent, including both young and older women, who were trained to maximize the comfort of participants during the FGDs and PM activities, including their differing age groups. All KIIs were conducted in English, while all FGDs and PMs were conducted in the primary local language spoken by the adolescent girls and women in each context (Rohingya, Burmese, Arabic). Tape-recording was not used so as to ensure the participants felt comfortable providing information on a sensitive topic (menstruation) but careful note-taking was conducted by the two team members, capturing both verbal and non-verbal responses during the KIIs, FGDs and PM discussions. All participants provided oral informed consent before beginning data collection.

All study procedures were approved by the Columbia University Institutional Review Board (IRB) and the IRC IRB, and through a facilitated and systematic process of ethical review of the protocol by two local experts in each country.

### Data analysis

Two members of the research team reviewed all of the qualitative data transcripts, (KII, FGDs, PM) with the data analyzed using Malterud’s ‘systematic text condensation,’ a descriptive and explorative method for thematic analysis [17]. This approach utilizes a series of steps, including: a) broad impression, b) identification of the key themes, c) condensing the text from the code and exploring meaning, and d) synthesizing. The key themes identified from the data were shared with the entire research team for additional validation and discussion. In the following section, we present the major analytical themes found in this analysis and excerpts that best illustrate the recurrent descriptive codes under each analytical theme.

## Results

Four thematic areas emerged from the analysis: 1) Changes in menstrual hygiene practices among girls and women after displacement; 2) inadequate safe, private spaces for changing menstrual materials and disposal; 3) insufficient guidance provided by response staff to beneficiaries on the basics of MHM; and 4) inadequate cross-sectoral leadership and coordination on the content and timing of MHM responses. Both similarities and differences were identified in the experiences of girls and women in the two settings, illustrating the importance of adapting each MHM response to cultural and local contextual realities.

### Changes in menstrual hygiene practices among girls and women after displacement

The types of materials (e.g. disposable pads, reusable pads, cloths, etc.) used by displaced girls and women to manage monthly blood flow tended to change after displacement. The changes appeared to be influenced by one, the types of materials that were provided or otherwise available, and two, the ease of their ability (or not) to wash and dry reusable materials, or dispose of disposable materials.

#### Types of materials used

The types of menstrual materials that girls and women were using in Myanmar and Lebanon was found to be influenced by four things: 1) what was included in Non-food Item (NFI) distributions; 2) the frequency of NFI distributions; 3) girls’ and women’s mobility; and 4) the existence of personal funds.

In Myanmar, many girls and women reported a reliance on blanket NFI distributions that included disposable sanitary pads and other supportive materials, such as underwear and soap. The KIIs with emergency response staff revealed how during the acute phase, assumptions were made about the materials that girls and women would prefer, and would be easier to use in the particular displacement context. As one Hygiene Promotion Manager explained:

*… they told us they were using cloth but the same cloth was difficult to come by and we were worried where they would dry the cloth and taking into the consideration that there was no privacy.*

*So that problem was solved when we shifted to sanitary pads and not the cloth….*

The use of disposable pads was, however, described as new, particularly for those coming from rural areas. As one female Muslim caseworker in Myanmar explained during a KII: “At first many of the women used cloths but now all of them use the [disposable] pads provided*.”* She suggested that although many had previously used cloth to manage menstruation, they now preferred disposable pads. A key reason was that disposable pads had built confidence around preventing leakage and increased their ability to engage in daily activities of living. As one adolescent girl explained, “When I have [disposable] pads, I will go outside but when I only have cloth, I will not*.”* Many girls reported saving the disposable pads for the times when they would be outside the house and needed product reliability.

In Lebanon, in contrast, many girls and women reported that pre-displacement they had already used disposable pads, and thus continued to prefer them. However, NFI distributions were reported by both beneficiaries and staff in Lebanon to have been sporadic throughout the response, and more recently to have only targeted adolescent girls at protection centers. Most refugees described not having received a distribution with sanitary supplies in over a year.

The frequency of NFI distributions and availability and affordability of other options were all found to influence the types of menstrual materials used. Given the limited nature of the distributions in Lebanon, the comparatively higher socioeconomic status of many Syrian refugees (as compared to Myanmar IDPs), and the presence of functioning markets in the urban context, girls and women generally sought out menstrual supplies in local stores. As one older Syrian woman explained, “we get them [disposable pads] in the pharmacy or supermarket. They are everywhere now.” In Myanmar, accessibility to the market and financial resources were more limited, and many girls and women reported to be reliant on NFI distributions for sanitary materials. Distributions were at best monthly and then bi-annually and explained by staff as dependent on the organization responsible for NFIs in a given camp. The protracted nature of the Myanmar emergency and the limited funding were also described as impacting distributions, and sustainability in both sites. As one reproductive health staff member noted:

*…I understand the preference for disposables [by girls and women], but it seems like there is no plan for sustainability and whether these people are going to be in the camps forever… the sanitation facilities are now being set up one way but what happens if they then must go back to cloth?...*

Furthermore, the infrequency of distributions of MHM supportive supplies, especially laundry soap, created further challenges for girls and woman in terms of their ability to sustain the use of reusable materials, thus heightening their preference towards easier maintenance disposable options*.*

From the girls and women’s perspective, the irregularity of the supplies influenced their practices, with some IDPs in Myanmar describing reverting back to cloths due to a shortage of disposable pads. They also described the trading or sharing of menstrual supplies. As a WASH actor described:

*…although the PDM [post-distribution monitoring] showed minimal selling of menstrual materials, there was some. In general, two families would take their kits and sell one of them; then sharing the supplies in the one kit across the females in their families….*

Observations by the research team of the very limited local market in Myanmar indicated some sales of NFI sourced disposable pads, which emergency staff explained happens due to economic struggles faced by families in the camps. In Lebanon, the majority of displaced Syrian girls and women indicated that although they preferred disposable pads, such products were expensive in the local markets. This forced a shift to makeshift methods. As one WASH response staff explained*, “*They have coping mechanisms for when they can’t afford them. For example, the mothers will just use tissues for their menstruation and then share the pads with their daughters. Or, if none of them has them, they will all just use tissues*.”*

The mobility of the displaced also appeared to influence the type of material used. In Lebanon, discussions with both beneficiaries and staff revealed how refugees could access local markets. In contrast, the IDPs in Myanmar had very restricted movement. As one Hygiene Promotion Manager explained, “They have no livelihood so they do not have anything [menstrual materials]…because of restricted movements, the Muslim IDP can’t access these materials in town or even have the money to buy it [pads].”

#### Constraints of the environment on material use

The environment was also found to influence girls’ and women’s choice of materials and related menstrual practices due to limited privacy and a lack of disposal options available. Beneficiaries in both sites reported living in environments with diminished privacy for washing and drying of menstrual materials (e.g. reusable pads, cloths, underwear), and for disposing of pads and other used menstrual materials. Girls and women in both sites also indicated an increased preference for disposable pads due to the privacy and logistical challenges around washing and drying reusable materials, such as cloth. As one displaced woman in Myanmar explained:

*…the houses are too small and don’t allow for privacy. It is one room for the entire family. The size of the house and the lack of separation from the men and boys is a problem….*

For girls and women in Myanmar who continued to use cloth, laundering practices included hiding damp menstrual cloths underneath existing clothing or mattresses to dry. Such practices translated into longer drying times and the wearing of damp cloths, causing discomfort and irritation.

The space constraints in both emergency contexts also influenced disposal practices for menstrual waste, as indicated in the PM activities, with disposal of menstrual materials impacted by local beliefs and taboos. This was reflected in Myanmar where large rubbish bins existed as part of the waste management system, but many girls and women reported a preference for burying used materials, as they had done pre-displacement. However, the restricted size of the camps and the lack of privacy made the task of burying very difficult. Therefore, many reported either going out after dark or before dawn to bury their materials, which was problematic given the flood-prone nature of the site. Alternatively, they dropped used materials directly into latrines, which was considered both convenient and discreet. Such practices had negative consequences on the lifespan of the latrine infrastructure, and were further complicated by gender dynamics and taboos. As a WASH engineer explained:

*… Women were putting the pads down the latrines and it was clogging the pipes. They would take sticks to try and force the pads down and this was creating problems. Also, because it was men doing the desludging, the men would refuse to take out the pads….*

Select WASH actors advocated for the usage of waste bins placed directly inside the latrines. However, these efforts were often met with resistance from the beneficiaries due to the strong cultural taboos and perceived humiliation regarding the prospect of others being able to see their used (bloodied) pads in the bins. Such issues were exacerbated by the lack of enforcement of gender segregation of toilet facilities, making girls and women even more uncomfortable. As one older Muslim IDP explained, “I prefer to bury because someone can see it if they throw it in the latrine; and men are using the female latrines.”

Syrian girls and women living in the informal settlements also reported that they now shared toilet facilities with several families. These toilets were reported to be cramped, dirty and lacking in separate space for disposal. Many described how they carry dark colored plastic bags for discreetly putting the used materials in, to be later disposed in the household trash.

### Inadequate safe, private spaces for changing menstrual materials and disposal

In both sites, girls and women described challenges in finding spaces to safely and privately change their menstrual materials, clean themselves and dispose of menstrual waste. In both Lebanon and Myanmar, household shelter structures were frequently shared with family members (and non-related individuals or families), and lacking in walls or separation. For the IDPs in Myanmar, the shared latrines were collectively described as being unsafe, uncomfortable and dirty. Girls and women described a lack of enforced gender segregation, large gaps in the bamboo walls permitting visibility, and an absence of locks on the doors. This resulted in many girls and women experiencing anxiety regarding the potential for “peeping toms” or intruders while using these facilities. Despite the limited single room structures provided upon arrival to the camps, some families had constructed small washrooms as add-ons to their shelters as an alternative solution. These were reported as more appropriate locations for changing menstrual materials, especially during the night. Most bathrooms in the Myanmar camps and informal settlements of Lebanon also lacked a water source inside or convenient to the latrines. This was especially challenging for the Syrians living in informal settlements, who expressed desires for the provision of pre-wet napkins or baby wipes to be included in distributions to help address these hygiene issues. Persisting cultural practices of anal cleansing found in the Myanmar camps however made the issue related to having water in the latrines less pressing as it was more habitual for the population to bring a water vessel into cubicles, regardless of the water source proximity to the latrine.

During both the PM and FGDs, latrine accessibility during the night was also considered problematic in the camps, due to inadequate lighting on pathways and at toilets, fears of violence or of ghosts. Many girls and women opted to wake up very early (4 or 5 am) to use the latrines. Although this required walking outside in the dark across long distances, many believing that it reduced the risk for intruders in the latrines, contact with males and the absence of long queues that were common during daytime.

Similarly, in Lebanon, some refugee girls and women living in informal settlements encountered considerable challenges to finding private spaces within their shelters (i.e. tents, tarp structures, dilapidated or unfinished buildings) to change their menstrual materials, leading to the decision to change in the toilets despite how cramped and unclean they were. As one adolescent girl explained during the PM activity:

*…The walls that make the tents and separate them are normally just blankets, plastic sheeting and transparent. Someone from the outside can see you in there; if you undressed or don’t have your veil on, someone can see you….*

**Fig. 1:**
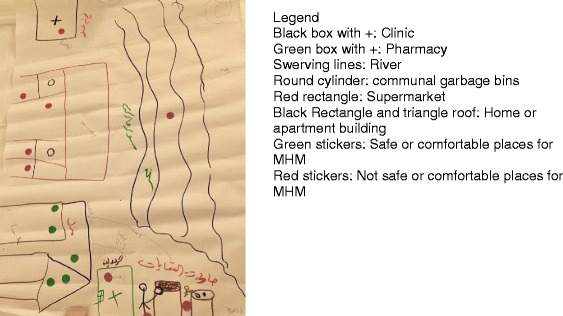
Example participatory map developed by Syrian girls living in a host community in Lebanon. Legend: Black box with +: Clinic, Green box with +: Pharmacy, Swerving lines: River, Round cylinder: communal garbage bins, Red rectangle: Supermarket, Black Rectangle and triangle roof: Home or apartment building, Green stickers: Safe or comfortable places for MHM, Red stickers: Not safe or comfortable places for MHM

The majority of girls and women indicated extreme discomfort at the thought of changing their menstrual materials inside their shelters, which was highlighted in both FGD and PM discussions (see Fig. 1). Although inadequate, shared toilets were still preferred for changing menstrual materials over a shared shelter. Latrine facilities were generally located around 10 ft from a household, and described as cramped, often lacking locks or doors and shared by several households. Despite the discomfort, most girls and women did not feel they had an alternative. As one woman explained: *“*I have just adapted. I have lived here for 3 years so I had to get used to it*.”* Many girls and women explained how they had adopted practices to reduce interactions with males when using the toilets, especially when menstruating. Some reported waiting until the early evening when men would generally leave the household, although this was reported to be challenging with heavy blood flow. Nighttime use of latrines also posed challenges, with many girls and women citing fears of violence, kidnapping or snakes. As one young Syrian woman explained: “The informal settlements are not secure, so we can’t go outside at night…because of the kids.” As a result, some households developed makeshift household toilets. As one Protection staff described:

*…they started making bathrooms right inside the tent. Hygiene wise, it’s not a great idea but security wise, it is. I saw many of them put it right next to kitchen so there is a smell. But the women and girls think it’s a better idea than having to go use the ones outside.*

### Insufficient guidance provided by response staff to beneficiaries on the basics of MHM

The inadequate provision of basic MHM guidance through hygiene promotion during NFI or targeted distributions of menstrual supplies was reported by humanitarian response staff in both sites. One explanation heard repeatedly in both sites was that WASH and protection staff were not comfortable with the details of MHM and how to deliver guidance in a culturally appropriate way. One adolescent girl-focused Protection officer described this challenge in Lebanon, noting how “it is very challenging to give this information [to girls] and training staff is also hard. The staff come from their same culture and they also shy, especially when girls ask questions.” The provision of information about puberty (and menarche) to pubescent girls (and their caregivers) was also found to be lacking in Education and Protection programming.

Girls, women and staff in both sites described a gap in the provision of practical information about MHM. This was especially the case for girls and women living in the Myanmar camps, from more rural settings who switched from cloths to disposable pads after displacement. This included the importance of good hygiene practices and camp management’s preferred disposal practices (so as not to damage toilet infrastructure). As a Women’s Protection staff in Myanmar described:

*…They were never taught at first how to use the pads or how to dispose of them. The women who came from town [had] used pads before so when they moved here, they showed the people from villages how to use them. They need education on using the pads and disposal...for example, when they first got the pads, they just thought they were tissues*….

In both contexts, staff and beneficiary participants noted the importance of adapting such information to the literacy and cultural backgrounds of the population. Syrian refugees were found to have a wider range of educational backgrounds and varying cultural differences between the previously urban and rural populations, in contrast to the Myanmar IDPs, who were described as having low literacy levels. One suggestion made by a caseworker from the IDP population was that MHM guidance be developed that utilizes more visual or picture-based approaches and actual demonstrations to support educational efforts. Another challenge, unique to the Myanmar context, included the IDPs lack of connectivity to media, due to minimal access to electricity and media networks. A Reproductive Health staff described this challenge, explaining that “menstrual hygiene is so important because they [the IDPs] are so isolated. When we compare the [town] situation to the camps, they at least have the media or other things. But in the camps, they are not aware at all of these things.”

Regarding the provision of puberty and MHM guidance to pubescent girls and their caregivers, varying opinions surfaced among response staff in the two sites about whether such information belonged in acute versus protracted response contexts. This debate also considered the funding implications, as one education adviser working in Myanmar explained:

*…the challenge for education in emergencies, as a sector, is that the focus is on primary education which here in Myanmar is ages 5–11; so most girls are not menstruating yet. The funding for older kids generally comes later, so the opening to discuss MHM is more limited in the acute phase….*

In Myanmar, many girls indicated they had not been aware of what menstruation was prior to their first menstrual period, causing distress and embarrassment at the onset of bleeding. Adolescent girls described how after sharing their discovery of bleeding with a family member, usually mothers, sisters or sister-in-law, they gained an understanding of how to manage menstrual blood. The majority of adolescent girls and women reported that they had not received any form of education on the biology of menstruation or how it pertains to their reproductive health during their time in the camps or pre-displacement.

In contrast, the Syrian refugee adolescent girls and young women in Lebanon were more likely to report having some basic knowledge about menstruation prior to their first period. They described having learned about it in schools or from their families. As one Protection staff explained:

*Many girls have a lot of information, but it is not correct. The other girls have no idea what we are talking about. It differs a lot between girls...and there are a lot of misconceptions though, like about not being able to shower during your period.*

Such misconceptions could have been linked to actual cultural beliefs among the population, or possibly just adolescent girl’s misunderstandings about menstrual hygiene; regardless the need was indicated for the provision of more accurate information. Many Syrian refugee mothers also described feeling inadequately prepared to provide accurate education on menstruation to their daughters, sharing their own discomfort and lack of knowledge. As one mother explained:

*My daughter needs more information, but she’s still a child so I don’t know how to tell her. I was shy and embarrassed; my mother didn’t prepare me so I don’t know how to prepare her.*

Several women advocated for the provision of adult education to be provided to them on topics related to menstruation, to guide and support them in their efforts to communicate with their daughters on the topic, in addition to educating them on other relevant reproductive health topics, such as menopause. As one Protection Manager explained, “This is a subject [menstruation] that women ask about a lot and we don’t have materials to give to them.”

The experience of displacement also appeared to reduce some previously experienced taboos that menstruation introduced into girls and women’s lives. Both Syrian girls and their mothers identified numerous restrictions they had been taught to respect when menstruating, such as on bathing, cooking, praying, interacting with males and eating specific foods before displacement. However, displacement conditions made many of these restrictions increasingly difficult to adhere to, suggesting the possible diminishment of such taboos. Although the girls and women in the Myanmar camps also reported some restrictions related to menstruating status, there was no mention of a shift in such taboos subsequent to displacement.

### Inadequate cross-sectoral leadership and coordination on the content and timing of MHM responses

In both emergencies, it was found that a range of different sectoral actors (WASH, Protection, NFI’s) were involved, to varying degrees, with the introduction of MHM activities, primarily the distributions of materials. However, the KIIs with humanitarian response staff in both settings revealed a lack of buy-in and consensus on the best practices and strategies for implementing an MHM response. Staff across various sectors explained how the absence of detailed guidelines or strategies for introducing MHM, both internally within organizations and externally in broader sectoral guidelines (e.g. Sphere), hindered their ability to respond and to effectively collaborate. As one reproductive health advisor in Myanmar explained:

*When there was flooding again, we saw multiple issues that came up with the distributions of kits, including dignity kits…What are we giving? What are the standards? How often do we supply them? These are unanswered questions.*

In addition to the expressed uncertainty about effective approaches for the provision of sanitary materials (e.g. hygiene kits, dignity kits) to a given population within a given context (e.g. floods, conflict), there was found to be a lack of consensus or familiarity with what comprises the key elements of a complete MHM response.

Most respondents, regardless of their sector, when asked about the existing MHM response would articulate the importance of the provision of supplies. Additional prompting was often needed to capture their perspectives on the design of supportive facilities (toilets, bathing spaces), supplementary materials needed for washing and drying (e.g. basins, laundry soap), appropriate menstrual health and hygiene education, and methods for disposal and waste management. Related, there was found to be a lack of clarity surrounding the appropriate timing of an MHM response and the primary response, which lacked reflection or time dedicated to exploring the local population’s menstrual practice preferences (e.g. reusable materials versus disposable pads) or the cultural or social considerations that might influence its ultimate success. Concerns were also identified regarding a sector’s willingness to initially address MHM given competing priorities. As a one senior protection actor working in Myanmar explained:

*…WASH now has become broad. They will be looking at menstruation, gender issues, water. They embrace a lot things but in the acute phase, for WASH to concentrate on these other issues, is rare. They will usually degrade them to a later phase. Menstrual issues will come later—unless you have a [WASH] manager who is really into gender issues….*

The choice of materials was also found to not always be sufficiently coordinated with the waste management systems being implemented. This included attention to disposal systems, the impact of material selection on sanitation infrastructure, cultural beliefs surrounding disposal, challenges related to discreetly washing and drying reusable materials, the role of menstrual hygiene education to support these efforts, and waste management. Sustainability concerns also arose in relation to waste management, as one Hygiene Promoter described in the camps in Myanmar in relation to concerns about diminished response funding, “if it [the emergency] continues, who is going to be responsible for picking the pads? Or running the incinerators?*”*

Another important factor articulated in both sites as hindering an effective MHM response was a lack of clarity about which sector had the responsibility (and funding support) to lead and coordinate a cross-sectoral MHM response beyond the provision of supplies. This includes explicit directions or guidelines that place these responsibilities within a specific lead sector with other supporting sectoral actors. Although many respondents suggested that the WASH sector does and should play a central role in leading MHM efforts, there was concern expressed for the discomfort that male engineers in particular might feel addressing MHM with beneficiaries. A number of respondents suggested that a critical role in an MHM response thus exists for the Protection sector, and in particular the GBV sub-cluster, whose staff are frequently closer to women and girls. However, there was found to be uncertainty about specific roles for the non-WASH sectors and information sharing to enhance MHM response efforts. As a Women’s Protection staff in Lebanon explained:

*We wanted to collaborate more with them [WASH actors] and to get them to take into consideration our referrals. We do GBV assessments – in this location, the women and girls are not safe. There are many GBV cases at the latrines and the latrine is always one of the main location or reasons for it…they need to build more toilets, and can we have locks? Can they be divided [by gender]?*

In particular, the Health sector requires improved clarity. For example, in both Myanmar and Lebanon, Health Sector staff acknowledged that their sector should have roles in supporting MHM, but were unclear on the specifics. A Senior Reproductive Health Adviser in Lebanon articulated this concern, explaining “We need to figure it out where MHM fits… is it SRH (Sexual and Reproductive Health)? Maybe…but I don’t know. I think for us, as part of awareness we do talk about hygiene through our partners...but not necessarily menstrual hygiene.” The delineation of roles for each sector could mitigate this confusion, enable a more timely and comprehensive response, and reduce the potential for overlap.

## Discussion

The findings from the qualitative assessments on menstrual management conducted in two differing emergency contexts, provided valuable insights into the range of key issues related to MHM in humanitarian response settings. An “MHM response” was primarily understood as, and focused on, the provision of sanitary materials, rather than a mainstreaming of MHM into various relevant sectoral response efforts (e.g. WASH, Protection, Health, Education). Other key findings included inadequate consultation with beneficiaries on menstrual practices, insufficient coordination between sectors, lack of clarity on leadership on MHM, and insufficient existing guidance on appropriate and effective MHM interventions in emergency contexts. However there was at the same time a great interest demonstrated in understanding what an improved MHM response would include among humanitarian response staff, and a willingness from beneficiaries in both sites to share their MHM needs.

In both sites there was found to be a need for improved understanding of beneficiary MHM practices at the onset of an emergency. Many of the staff working in the two emergencies, as well as the beneficiaries themselves, indicated inadequate consultation had occurred with girls and women about their preferences and practices. This might have resulted from staff discomfort with the topic, lack of prioritization of MHM as an important issue during the emergency, or other priorities that were deemed more critical during the initial acute phase. However basic consultations with girls, women and local staff are essential given the likely variations in menstrual practices even within a specific population [13]. Many response staff in both sites articulated a lack of familiarity with the details surrounding how beneficiaries were managing menstruation. One proposed solution for future emergencies was the establishment of a global or national level database which would be tasked with gathering basic information on local women’s menstrual practices and beliefs prior to the onset of an emergency, especially in regions or countries considered disaster or conflict prone. This information would then be readily available to inform response efforts during the acute phase, when gathering such information is perceived as challenging given the sensitivity of the topic and competing priorities. Such an approach would pre-position knowledge about cultural practice, however there are also approaches for making the “best available option” choices in the acute phase in the absence of such information.

Improved consultations with girls and women at the onset of an emergency is likely to illuminate these key issues earlier, thus improving the quality of the response. In addition, for situations where the displaced are living in peri-urban or urban informal settlements (including tent-like dwellings), there may arise unique challenges for WASH actors in addressing their MHM needs. WASH programs have in some contexts experienced challenges identifying and supporting sanitation infrastructure in informal settlements [18], given that many shelters were often located on privately-owned or deserted properties.

This study also highlighted a need for improved cross-sectoral coordination on MHM. Although numerous mentions were made of the importance of the WASH and Protection sectors working together to understand girls’ and women’s MHM needs and challenges, there was much less mention of specific examples of ways MHM was integrated into other sectoral responses. For example, this might include WASH actors consulting with Health actors to ensure female friendly WASH facilities exist at health facilities. Or collaboration between WASH, Protection and Shelter actors given the finding from the Myanmar camps that girls and women prefer changing menstrual materials in informal washrooms attached to their one-room household structures. Such spaces were perceived to be safer, accessible at night, and more private. This example highlights the importance of directly consulting girls and women, as assumptions are frequently made about their supposed preference to manage menses within latrines or communal bathing facilities [19]. along with the importance of cross-sectoral collaboration. At the same time, resources and funding often dictate the limits of what can be implemented, so although household toilets are preferable for MHM, they may not always be realistic in a given response.

There was also revealed to be a lack of certainty about the appropriate sector to lead on MHM in a given emergency. Although there was found to be general consensus in both contexts about the importance of WASH as the lead sector in collaboration with the Protection sector, many response staff highlighted the challenge of how to respond effectively on MHM if there is not a strong WASH presence in a given emergency. This was found to be the case in Lebanon, where WASH actors have had a more minimal role than they might have had in a camp setting. In such scenarios, there is a need for another key sector to take on a leadership role to fill this gap on MHM. In the case of Lebanon, Protection was seen as the most viable leader for leading and coordinating a response on MHM, however learning from on-going and future emergencies will help to provide additional examples of effective leadership on MHM by WASH, Protection or other sectors.

Another key issue that emerged in both Myanmar and Lebanon was the challenge of regularly providing sufficient amounts of MHM supplies. This is especially the case for distribution dependent populations, like the IDP camps in Myanmar, where the populations have minimal ability to generate livelihood or access markets. A sustainability challenge was also highlighted in relation to the impact local menstrual practices may have on the lifespan of WASH infrastructure, if not adequately addressed by the WASH sector (both software and hardware interventions). During the design phase, WASH actors must also think about how girls and women may be driven to putting used materials, such as disposable pads and cloth, directly into toilets in order to avoid serious implications for the longevity of these facilities, particularly when they are communal and already prone to filling up more quickly or to clogging. The findings also identified concerns about how menstrual waste operations, such as sanitation workers assigned to collect waste and manage incinerators, could continue to operate if funding was not sustained. The concern was particularly relevant for the topic of MHM given the many taboos that exist around the handling of menstrual waste, and the likelihood that only paid staff would be willing to manage waste disposal systems.

The diversity of these emergencies, and the use of qualitative research methods, mean that this learning is not generalizable to all types of emergencies. Although the two assessments aimed to gather insights from a range of actors and individuals, there are important limitations to note. One, given competing priorities of those living and working in both emergencies interviewed, these assessments were necessarily rapid and given the assessments were qualitative, the findings are not necessarily reflective of the entire population. Two, the role of local IRC staff in assisting with in-country logistics, with IRC also a key service delivery organization for these populations, it is possible that bias existed in the beneficiary responses provided despite efforts to assure the confidentiality of the information collected. Three, the girls and women interviewed were those who are in contact with humanitarian agencies and so the data collection did not include those not in contact who may be living in more dire circumstances.

Lastly, although not explored during this study, the growth of new programming strategies, like the introduction of cash transfers and vouchers for the delivery of hygiene-related aid, including menstrual items [20], is similar to other interventions in that it creates new areas necessitating improved consultation with beneficiaries. Such efforts have to date inadequately investigated the impact of such innovations on girls and women’s ability to access menstrual hygiene items. This is especially important as women often have less decision-making power in the household, and in the face of rising household expenses and competing live-saving priorities, may have less ability to prioritize MHM supplies with cash or vouchers [21].

## Conclusion

The learning from these two assessments highlights the need for the humanitarian response community to better understand the holistic definition of an MHM response, one that includes the provision of female friendly WASH facilities, appropriate information, and supportive menstrual supplies. This includes the importance of clear, designated leadership on MHM in a given emergency, enhanced cross-sectoral collaboration, and the integration of MHM into other relevant sectoral response efforts. The most essential component remains continuous consultation with adolescent girls and women.
